# Putting data to work for precision medicine

**DOI:** 10.1016/j.xcrm.2023.101090

**Published:** 2023-06-20

**Authors:** Andreas Bjerrum, Ulrik Lassen

**Affiliations:** 1 Department of Oncology, Copenhagen University Hospital, Rigshospitalet, Blegdamsvej 3, København, Denmark

## Abstract

We discuss the One Stop Shop for Clinical Research (OSCAR) project that links clinical data, patient-reported outcomes, genomic data, and health registry data, employing a rigorous data privacy protection technology, to provide insights into treatment efficacy and safety and serve as a comparator for single-arm trials. This could inspire further initiatives to advance precision medicine.

## Main text

Water, water everywhere and not a drop to drink… –Samuel Taylor Coleridge (“The Rime of the Ancient Mariner”)

Like water, personal health data is a valuable natural resource. It has abundant reservoirs in both public and private healthcare systems. Recent advances in health technology, human genetics, and our understanding of the molecular basis of disease continue to contribute significantly to the supply of personal health data worldwide.

However, like the water in Coleridge’s poem, the ability to use this critical resource in a form that will sustain the development of precision medicine remains elusive.

Healthcare providers and clinical researchers alike need reliable and legitimate access to personal health data from a variety of sources so they can personalize, utilize, and evaluate the benefit of treatments for individual patients and target diseases.^1^ Key roadblocks to personal health data access include a lack of healthcare infrastructure, enabling regulation, and dedicated financing.

In Denmark, an ongoing study focuses on a sustainable solution to address the personal health data access challenge to advance precision medicine.

### Reconstructing individual health trajectories for people living in Denmark

Rapid advances in biomedical research, particularly within the fields of genomics, transcriptomics, and metabolomics are transforming our understanding of the complex nature of human biology and pathology. A healthcare infrastructure that secures the systematic collection of “omics” data in a validated and structured manner that is fully interoperable with national disease registries would allow researchers and clinicians to optimize the potential value of health registries to advance precision medicine.

Denmark has one of the best national systems in the world for collecting health data.^2^ In recent years, the country’s leadership has begun to realize the importance of utilizing its national health data more efficiently, and in 2021, the Danish government published a new Life Science Strategy. The strategy kicks off a national commitment to improve clinical conditions for research and development activities that encompasses improving access to health data to advance more cost-effective precision medicine.^3^

With the Life Science Strategy as the backdrop, Copenhagen’s major hospital center, Rigshospitalet, together with private and government partners, started a collaboration with the objective to build a comprehensive ecosystem for personal health data access called the One Stop Shop for Clinical Research (OSCAR). In addition to seven public and private partners, the OSCAR project is informed by a public advisory board with 13 members and a private advisory board with 22 members.^4^

In Denmark, regulation of who should be able to access health data, and for which purposes, is intensively debated, which has stimulated regulatory changes.^5^ A work package in OSCAR aims at providing clarity on the rules and regulations for data sharing as well as stimulating legislative processes to enable better use of health data for research purposes.

As a public-private partnership, OSCAR has secured access to a wide range of data sources in Denmark using an encryption technology that enables statistical analysis without compromising data privacy. Data sources in the OSCAR project include comprehensive population-based registries that hold information about health, socioeconomic, and employment status, as well as electronic health records (EHRs) together with patient reported outcome measures (PROMs) and advanced genomic data for a defined cohort of participants in the Copenhagen Master Observational Trial (C-MOT).

### C-MOT protocol as proof of concept for OSCAR

The master observational trial protocol is a trial concept that seeks to combine the depth of molecularly based master protocols (e.g., basket and umbrella trials) with the breadth of real-world data (RWD).^6^

The C-MOT employs this concept to collect data prospectively on all patients diagnosed with breast or lung cancer who receive treatment at two cancer centers in the Capital Region of Denmark, Rigshospitalet and Herlev Hospital, over a 30-month study period. To reflect the real-world setting, all patients can be included regardless of treatment strategy and ECOG performance status, provided that the patient has a life expectancy of at least 3 months.^7^

In Denmark, public hospitals are the only providers of oncologic treatments for breast and lung cancers, which ensures robust long-term follow-up for patients. The data being collected in C-MOT will include PROMs, EHRs, germline whole-genome sequencing (WGS), and tumor WGS and RNA conducted at baseline and progression, thus following any subsequent change in cancer genetics as a response to the treatment during the study. The quality of real-world evidence (RWE) from these diverse sources will be secured using standardized clinical training protocols and controlled terminologies to govern data collection.

By collecting longitudinal data for all patients and combining them with individual molecular data, the goal is to map a comprehensive health journey for each patient by linking data sources.

### Combining genomic and phenomic insights for further research

Cancers often contain multiple gene variants, and interpretation of the clinical relevance of one variant must also consider the effects of other coexisting mutations. Even if the clinical relevance of one mutation is well documented for one tumor type, the clinical relevance of the same mutation in a different tumor context may be less clear.^8^

C-MOT gathers comprehensive longitudinal, phenomic data from EHRs, including detailed information about dosing of anti-neoplastic drugs (e.g., dose modifications and adverse events), cancer supportive care drugs, comorbidities, biochemistry, microbiology, and pathology, as well as repeated measurements of vital signs and radiology workup. We use the Observational Medical Outcomes Partnership Common Data Model^9^ to standardize the structure and content of the EHR data to enable efficient analyses and data collaboration with other sites (e.g., federated data analysis).

By combining standardized information from PROMs and this extensive “phenomic profile” with genomic data for each patient, the C-MOT will provide valuable insights into the complex interplay between germline and somatic variants and their subsequent impact on the efficacy and safety of available treatment options, whether standard-of-care or new treatments in development.

The availability of EHR data for the C-MOT project is made possible after significant public investments in a new software application that was rolled out in 2016 to cover all hospitals in the Capital Region of Denmark. While enabling extraction of EHR data, implementation of the software was complex and led to dissatisfaction.^10^ Further, attempts to repurpose clinical data for administrative or research purposes may drain resources from clinical care, where additional data needs consume clinical resources.^11^ The C-MOT relies on EHR data that is routinely entered and does not require any additional data entry. However, analyses of preliminary EHR data in C-MOT have exposed data weaknesses and deficiencies in such areas as the systematic documentation of treatment toxicity and ECOG performance status, as well as systematic radiological documentation of the Response Evaluation Criteria In Solid Tumors. We are striving to enhance structured documentation for these areas because we believe that the additional efforts will be valuable for daily clinical practice in addition to enhancing data quality for research. Institutions investing in EHR infrastructure should evaluate the system’s ability to provide structured information on treatment toxicities and responses.

### Leveraging rigor of RCTs to reap the benefits of RWE

Both the European Medicine Agency and the US Food and Drug Administration (FDA) have increased their focus on RWE over the last 5 years.^12^^,^^13^

The US FDA has recognized that RWE of sufficient quality can supplement data from randomized controlled trials (RCTs) to paint a more complete picture of how a health intervention works in a broader patient population. Because of its plentiful nature, the vision is that RWE can fill the knowledge gaps that exist between the limited number of patients in clinical trials and the relatively larger number of patients in the actual practice of medicine. This is even more relevant for uncommon cancers and other rare disorders, where patient numbers are few. In this context, RWE can play an essential role to expedite the availability of safety and efficacy data and/or serve as a comparator for single-arm trials of experimental therapies.^14^

The FDA evaluates the quality of RWD based on how well the data collection effort controls for bias.^6^ For instance, the issue of inclusion bias is a consideration because some patients may not be offered entry, e.g., if the consultation time is too short or due to comorbidity or miscommunication.^15^ The process of obtaining informed consent to participate in C-MOT is delegated to all doctors, as well as selected nurses who are trained in the protocol. This means that inclusion can happen during both treatment and consultation sessions. Since participation in C-MOT does not require any extra visits, we believe that there is little additional economic or time burden for study participants, which will likely have a positive effect on participants’ willingness to join and remain enrolled for the full study period.

Study participants can install an app on their mobile device to answer the PROMs questionnaires, or they may provide their answer on paper if they prefer. Inability to complete the PROMs is not an exclusion criteria, which may reduce selection bias but at the cost of reduced completeness and possibly increased bias in the PROM data.

### Clearing a path for the OSCAR dream to become a reality

There is growing recognition in Denmark that clinical researchers need more open access to health data to advance precision medicine initiatives like the C-MOT. In turn, these research endeavors must fulfill GCP guidelines including transparent governance, institutional ethics review board oversight, informed patient consent, and secure data privacy protection.

While the C-MOT protocol has been approved by ethics committees, we seek to expand this approval even further to allow all data from the trial to be uploaded to a data lake hosted on the OSCAR platform. This would allow fellow researchers to not only access data from C-MOT but also cross link individual data from the C-MOT with the Danish public registries using Danish residents’ unique personal identification numbers within the same ecosystem.

Ultimately, our ambition is to ensure that all cancer patients treated at Rigshospitalet are included in a master observational trial and that the C-MOT will provide the needed knowledge and experience to the wider research community in Denmark and abroad with similar ambitions.

### One step closer to the promise of precision medicine

Precision medicine represents a paradigm in medical care that will require new ways of working, new laws governing health data access and utilization, a new scope for patient consent, and a new mindset for both public and private healthcare providers and research organizations, as well as the governing bodies that regulate them.

The end users of the healthcare system—patients and caregivers—see the value of precision medicine and have been advocating for it for a long time. It is the advent of genomic medicine and advanced information technologies that is helping to realize the promise of precision medicine to improve individual patient outcomes and increase the efficiency and value of the healthcare system overall.

With the OSCAR project, we have established a private-public collaboration to advance this paradigm shift by building an intuitive and legitimate entry point for accessing and combining registry data with genomic and clinical RWD to support quality research for the development, evaluation, and clinical application of precision medicine.
